# Aniseikonia and retinal morphological changes in eyes undergoing macular hole surgery

**DOI:** 10.1038/s41598-023-51032-0

**Published:** 2024-01-02

**Authors:** Asuka Takeyama, Yutaka Imamura, Taichi Fujimoto, Toshiya Iida, Yuko Komiya, Masahiro Ishida

**Affiliations:** 1 Department of Ophthalmology, Toho University Ohashi Medical Center, 2-22-36, Ohashi, Meguro-ku, Tokyo, 153-8515 Japan; 2 Department of Ophthalmology, Teikyo University School of Medicine, University Hospital Mizonokuchi, Kanagawa, Japan

**Keywords:** Retinal diseases, Predictive markers

## Abstract

Even after idiopathic macular hole (MH) surgery and with successful closure of MH, aniseikonia is a common postoperative symptom. We investigated the correlation of MH diameter, retinal displacement and retinal layer thicknesses with aniseikonia in 41 eyes of 41 patients undergoing MH surgery with internal limiting membrane peeling. Aniseikonia was measured with the New Aniseikonia Test. Retinal displacement (RD%) was defined as change of retinal distance between the temporal margin of the optic papilla and the intersection of the retinal vessels. Changes of thicknesses of the inner nuclear layer (INL%) and the outer retinal layer (OR%) were calculated. Aniseikonia improved postoperatively. Preoperative aniseikonia and their improvement at 6 months correlated with MH diameters (*P* = 0.004–0.046). Improvement of aniseikonia correlated with temporal RD% (*P* = 0.002–0.012). Improvement of vertical aniseikonia correlated with INL% at 2 weeks and with the nasal OR% at 1, 3, and 6 months (*P* = < 0.001–0.028). MH diameter and age were significant predictors for improvement of aniseikonia. The greater the temporal retina displacement, and the thinner the postoperative INL and OR, the greater the improvement of aniseikonia. MH diameter and age are strong predictors for improvement of aniseikonia after MH surgery.

## Introduction

Recent advances in vitreoretinal surgical techniques for the repair of idiopathic macular hole (MH) have improved anatomical success rates and visual outcomes^1–4^. However, even in cases of successful and uncomplicated surgery, visual complaints such as metamorphopsia and aniseikonia are common symptoms before and after MH surgery.

Aniseikonia is a binocular condition perceived by both eyes as a difference in the size of ocular image. Aniseikonia may be caused by changes of the density of photoreceptor cells due to the elongation or contraction of the retina associated with retinal disease, resulting in changes in the size of the image^5^, however how aniseikonia is generated in patients with MH remains to be elucidated.

We previously reported that the temporal retinal displacement due to internal limiting membrane (ILM) peeling in MH surgery correlates with the basal diameter of the MH^6^, and that the retinal displacement correlates with changes in the inner nuclear layer thickness (INL), indirectly proving our hypothesis that retinal displacement is caused by contraction of the optic nerve fiber^7^. We showed that the degree of metamorphopsia correlates not only with changes in INL but also with MH diameter (MHD), and that photoreceptor displacement is the most important cause of metamorphopsia^8^.

As a step to elucidate the mechanism of aniseikonia in MH, we analyzed the relationship between the degree of aniseikonia, MHD, change of retinal thickness and retinal displacement before and after MH surgery. In addition, we identified predictors for pre- and postoperative aniseikonia and its improvement.

## Methods

A consecutive series of patients with idiopathic MH who underwent pars plana vitrectomy and achieved successful closure of the MH in the Department of Ophthalmology, Teikyo University School of Medicine, University Hospital Mizonokuchi from June 2017 to June 2021 were enrolled in this retrospective study. This study was performed in accordance with the tenets of the Declaration of Helsinki and received approval from the Institutional Review Committee of the Teikyo University School of Medicine (No. 20-206). Due to the retrospective nature of this study and the anonymized nature of the image analysis and data, need to obtain informed consent was waived by the Institutional Review Board of Teikyo University School of Medicine. This research information was presented on our institutional website and all the patients were provided the opportunity to opt out of this study.

Patients who were followed up for at least 6 months postoperatively were included. Exclusion criteria included eyes with an axial length ≧ 27 mm, glaucoma, retinal vascular disease, uveitis, proliferative diabetic retinopathy, traumatic MH and secondary MH, a history of vitreoretinal diseases except MH or vitreoretinal surgery and eyes undergoing the inverted ILM flap technique. Eyes with anisometropia of more than 2.0 D at baseline and 6 months postoperatively, eyes that changed more than 2.0 D postoperatively, and eyes with diseases that can affect anisometropia such as MH, epiretinal membrane (ERM), rhegmatogenous retinal detachment (RRD) and cystoid macular edema in the fellow eye was excluded. The best-corrected visual acuity (BCVA) using a Landolt C chart were performed preoperatively and at 2 weeks and 1, 3 and 6 months postoperatively.

### OCT measurements

**Figure 1:**
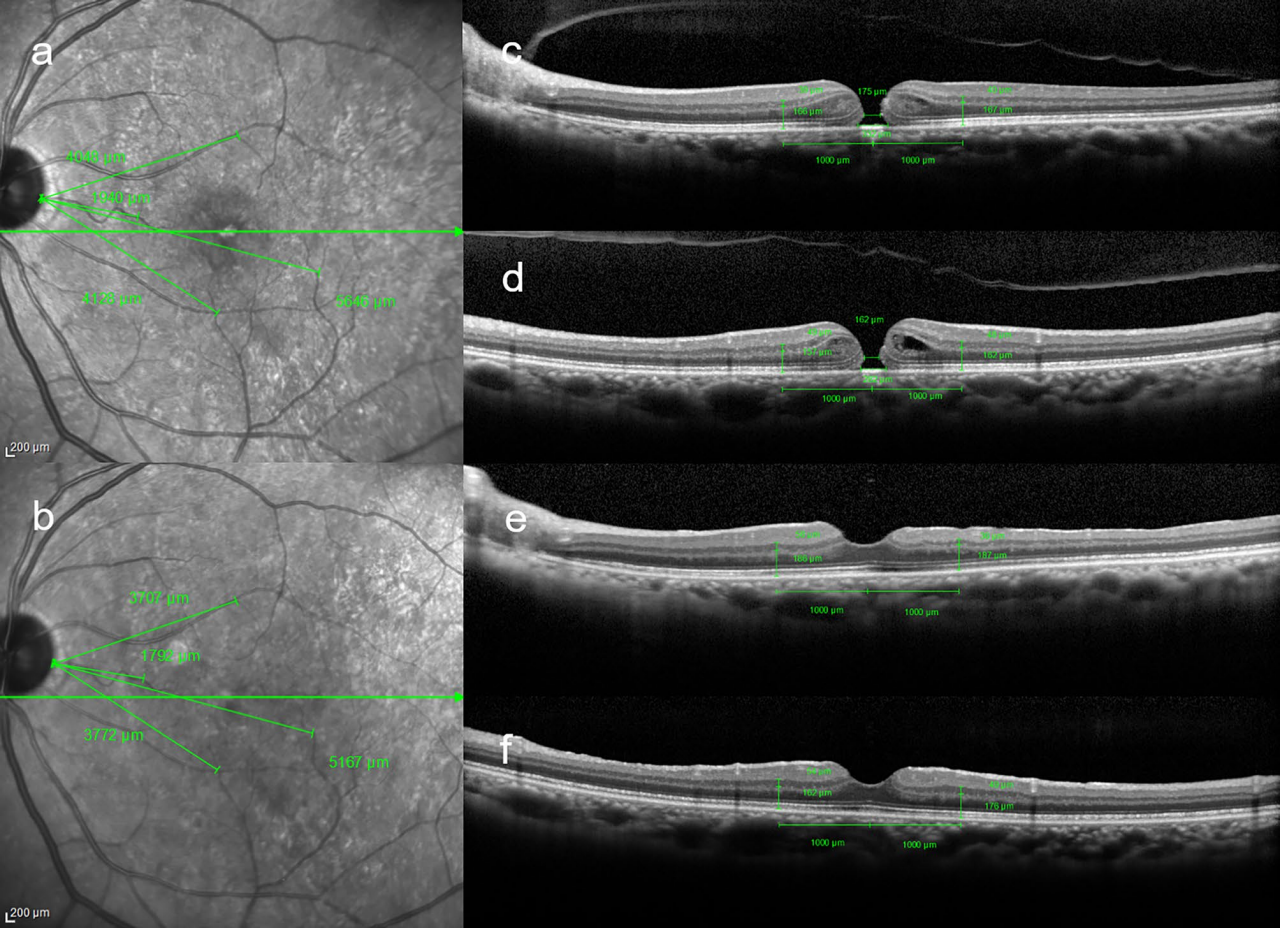
Measurement of parameters using spectralis optical coherence tomography before and after macular hole surgery. (**a**) Measurement of preoperative retinal distance. Using near-infrared imaging, four retinal vessel intersections or bifurcations were selected in the temporal, nasal, superior, and inferior regions of the early treatment diabetic retinopathy study subfield and the retinal distance between their points and the temporal margin of the optic disc were measured. (**b**) Measurement of retinal distance at 6 months postoperatively. The same method was used for the preoperative measurements. (**c**) Measurements of the macular hole diameter (MHD) and each retinal thickness. In horizontal scan images, the horizontal minimum and basal MHD, inner nuclear layer (INL) and outer retinal layer (OR) thicknesses 1000 μm away from the center of MH temporally and nasally were measured. (**d**) In the vertical scan image (superior retina on the right side of the image and inferior retina on the left side), the vertical minimum and basal MHD, INL and OR thicknesses were measured as in the horizontal image. (**e**) Retinal layer thicknesses on nasal and temporal sectors in the horizontal scan image at 6 months postoperatively. (**f**) Retinal layer thicknesses on the superior and inferior sectors in the vertical scan image at 6 months postoperatively.

Retinal distances and thicknesses of retinal layers were measured as previously reported using Spectralis (Heidelberg Engineering, Inc., Heidelberg, Germany) before and after surgery^7^. The methods of measurement for retinal distances, minimum and basal MHDs and retinal layer thicknesses are shown in Fig. 1. INL% and outer retinal thickness (OR) % were calculated as rate of changes of OR and INL after surgery in each sector. Retinal displacement (RD%) was calculated as rate of change of retinal distance after surgery in each sector.

### Aniseikonia measurement

The New Aniseikonia Test (NAT; Handaya Co., Tokyo, Japan) was used for quantitative evaluation of aniseikonia. The detailed procedure for NAT scoring is described in our previous reports^9^. Briefly, the degree of aniseikonia was measured by dissociating the two eyes using spectacles with a green filter in front of the right eye and a red filter in front of the left eye. The patients wore red–green spectacles and examined a book containing a pair of red and green semicircles at 40 cm distance. The diameter of the red semicircle on the left remains constant at 4 cm, while the size of the green semicircle on the right varies from 1 to 24% in 1% increments. The difference in the actual size of a pair of semicircles, in which the patient indicated two semicircles appear to be equal in size, represents the percentage of anisometropia. The NAT scores were examined for vertical and horizontal lines. Aniseikonia of more than + 2% was defined as macropsia and that of less than − 2%, as micropsia^10,11^.

### Surgical procedures

Pars plana vitrectomy with 25-gauge instruments combined with ILM peeling, air–liquid exchange and 20% sulfur hexafluoride tamponade were performed by 3 surgeons (M.I., Y.I. and A.T.). ILMs were removed by staining with the 10% triamcinolone acetonide or 0.25% brilliant blue G. ILM peeling was performed in all quadrants. With all the surgeons, ILM was peeled to the edges of vascular arcade, close to the optic papillary margin on the nasal side, and to the same or greater area on the temporal side. Phakic eyes over 50 years of age underwent phacoemulsification with intraocular lens implantation simultaneously with vitrectomy. The surgical procedures were the same as the procedures previously reported in detail^7^.

### Statistical analysis

The BCVA was analyzed on the logarithm of the minimal angle of resolution (logMAR) units for statistical analysis. A nonparametric test (the Shapiro–Wilk test) was chosen for evaluation, as our data did not follow a normal distribution. The Wilcoxon signed-rank test was performed to compare between preoperative and postoperative data. Correlations between NAT score, MHD, retinal thickness %, and RD% were determined using Spearman's rank correlation coefficient test. Multiple regression analysis was performed to identify predictors for NAT scores. SPSS software version 24.0 (SPSS, Chicago, Illinois, USA) was used for statistical analysis. A *P* value of < 0.05 was considered statistically significant.

## Results

Forty-one eyes of 41 patients were included in this study. The average age of patients at the time of surgery was 65.1 ± 6.4 years (range 50–78 years) and 23 patients (56.1%) were women. The average preoperative spherical equivalent was − 1.0 ± 1.8 D and absolute difference of spherical equivalent between both eyes was 0.3 ± 0.8 D. The average spherical equivalent at 6 months was − 0.8 ± 2.0 D and difference of spherical equivalent between preoperative and 6 months was 0.2 ± 1.1 D. The staging of MH was classified as follows: 17 eyes (41.5%) as stage 2, 17 eyes (41.5%) as stage 3, and 7 eyes (17.0%) as stage 4. Phacoemulsification with intraocular lens implantation simultaneously with vitrectomy was performed for all eyes. TA was used for staining for 17 eyes and BBG was used for 24 eyes during ILM peeling. Two eyes in which ILM was completely detached during surgery were included in this study, although an inverted ILM flap technique had been originally scheduled because the minimum MHD was greater than 450 μm.

After evaluating the mean values of vertical and horizontal NAT scores, 12 eyes (29.3%) had micropsia, 28 eyes (68.3%) had no aniseikonia, and 1 eye (2.4%) had macropsia preoperatively. Four eye (9.8%) had micropsia, 30 eyes (73.2%) had no aniseikonia and 7 eyes (17.0%) had macropsia at 6 months postoperatively.

The average horizontal minimum MHD was 266.7 ± 117.4 μm (range 68–584 μm) and the horizontal basal MHD was 651.3 ± 245.1 μm (range 109–1108 μm), whereas the average vertical minimum MHD was 241.6 ± 105.3 μm (range 43–513 μm) and the vertical basal MHD was 577.4 ± 227.4 μm (range 109–983 μm). The demographics and ocular characteristics are provided in Table 1.

**Table 1:** Demographics and ocular characteristics.

Age (years)	65.1 ± 6.4 (range 50–78)
Male/female	18/23
Axial length (mm)	23.9 ± 1.2 (range 21.8–26.7)
MH stage 2/stage 3/stage 4	17 (41.5%)/17 (41.5%)/7 (17.0%)
Average horizontal minimum MHD (μm)	266.7 ± 117.4 (range 68–584)
Average vertical minimum MHD (μm)	241.6 ± 105.3 (range 43–513)
Average horizontal basal MHD (μm)	651.3 ± 245.1 (range 109–1108)
Average vertical basal MHD (μm)	577.4 ± 227.4 (range 109–983)

The data are presented as the mean ± standard deviation.

*MH* macular hole, *MHD* macular hole diameter.

The average BCVA was 0.57 ± 1.20 logMAR units preoperatively, 0.38 ± 0.22 logMAR units at 2 weeks, 0.21 ± 0.21 logMAR units at 1 month, 0.10 ± 0.20 logMAR units at 3 months, 0.09 ± 0.20 logMAR units at 6 months postoperatively. The average BCVA for all postoperative visits improved significantly (*P* value range *P* < 0.001–0.001). Preoperative BCVA correlated with the mean of vertical and horizontal NAT score at preoperatively, 1 month and 3 months postoperatively (r = − 0.359, *P* = 0.021, r = − 0.433, *P* = 0.004 and r = − 0.421, *P* = 0.007, respectively). Postoperative BCVA correlated with the mean of vertical and horizontal NAT score at 3 and 6 months (r = − 0.355, *P* = 0.023 and r = − 0.396, *P* = 0.010). In other words, the better the postoperative BCVA at 3 and 6 months, the smaller the degree of micropsia.

### Time course of the average NAT scores

**Figure 2:**
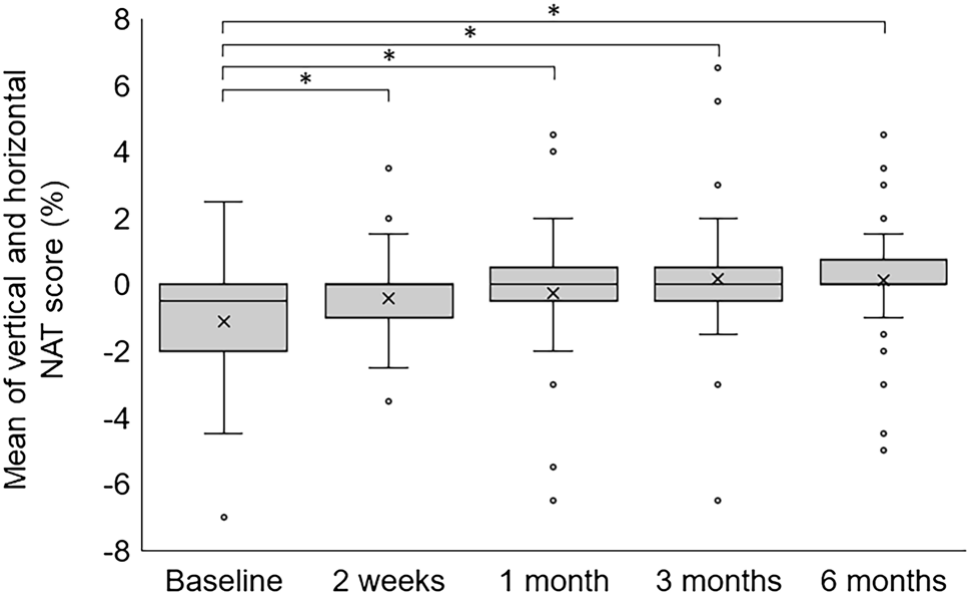
Box-and-whisker plot of the change in mean of horizontal and vertical new aniseikonia test scores after macular hole surgery. The top and bottom borders of the box indicate the 75th and 25th percentile, respectively. The crosse indicates the average score. The mean of horizontal and vertical preoperative vertical new aniseikonia test (NAT) score improved at all visits after surgery (*P* value range < 0.001–0.004). Significant *P* values are indicated by asterisk (*).

The average preoperative vertical NAT score was − 1.05 ± 2.00% which improved to − 0.02 ± 1.10% at 2 weeks (*P* < 0.001), − 0.12 ± 2.00% at 1 month (*P* = 0.001), 0.15 ± 1.83% at 3 months (*P* = 0.003) and 0.17 ± 1.71% at 6 months (*P* = 0.002). The average preoperative horizontal NAT score was − 1.22 ± 2.20%, which did not change to − 0.85 ± 2.07% at 2 weeks (*P* = 0.180) but improved to − 0.37 ± 2.34% at 1 month (*P* = 0.010), 0.17 ± 2.45% at 3 months (*P* = 0.001), and 0.32 ± 2.17%at 6 months (*P* < 0.001). The mean of vertical and horizontal the NAT scores after MH surgery are shown in Fig. 2. The mean of vertical and horizontal the NAT score was − 1.13 ± 1.94% which improved to − 0.43 ± 1.34% at 2 weeks (*P* = 0.004), − 0.24 ± 1.90% at 1 month (*P* = 0.001), 0.16 ± 1.98% at 3 months (*P* < 0.001) and 0.13 ± 1.81% at 6 months (*P* < 0.001).

### Correlations of NAT scores with MHD

Correlations of NAT scores with the MHD are shown in Table 2. The preoperative NAT scores showed correlations with MHD (r and *P* value range r = − 0.428 to − 0.313, *P* = 0.005–0.046). Horizontal NAT scores at 2 weeks correlated with the horizontal minimum MHDs, and vertical NAT score at 1 month correlated with the vertical and horizontal basal MHDs (r and *P* value range r = − 0.416 to − 0.326, *P* = 0.007–0.038).

**Table 2:** Correlations of new aniseikonia test scores with the diameters of the macular hole.

	Horizontal				Vertical			
	Basal diameter		Minimum diameter		Basal diameter		Minimum diameter	
	r	*P*	r	*P*	r	*P*	r	*P*
Baseline								
NATV	**− 0.418**	0.006*	− 0.282	0.074	**− 0.428**	0.005*	− 0.229	0.150
NATH	**− 0.394**	0.011*	**− 0.313**	0.046*	**− 0.407**	0.008*	− 0.299	0.058
At 2 weeks								
Difference of NATV	**0.386**	0.013*	0.102	0.525	**0.429**	0.005*	0.120	0.455
Difference of NATH	**0.407**	0.008*	0.048	0.764	**0.390**	0.012*	− 0.059	0.715
At 1 month								
Difference of NATV	0.159	0.320	0.138	0.391	0.100	0.535	0.018	0.913
Difference of NATH	0.124	0.439	0.236	0.138	0.087	0.587	0.151	0.344
At 3 months								
Difference of NATV	0.251	0.113	0.079	0.622	0.239	0.133	0.016	0.920
Difference of NATH	**0.460**	0.002*	0.224	0.159	**0.416**	0.007*	0.066	0.683
At 6 months								
Difference of NATV	**0.483**	0.004*	0.253	0.111	**0.413**	0.007*	0.162	0.311
Difference of NATH	**0.339**	0.021*	0.292	0.064	**0.322**	0.040*	0.167	0.296

Difference of NAT was defined as the difference between baseline and postoperative NAT scores.

Significant values are in [bold].

*NATV* vertical score of new aniseikonia test, *NATH* horizontal score of new aniseikonia test.

Significant *P* values are indicated by asterisk (*).

The difference in vertical and horizontal NAT scores at 2 weeks and 6 months correlated with the basal MHDs, respectively (r and *P* value range r = 0.322–0.438, *P* = 0.004–0.040). The difference in the horizontal NAT score at 3 months correlated with the vertical and horizontal basal MHDs (r = 0.416, *P* = 0.007 and r = 0.460, *P* = 0.002).

### Correlations of NAT scores with retinal displacement

Correlations of NAT scores with RD% are shown in Table 3. The preoperative vertical NAT score correlated with the temporal RD% at all visits postoperatively (r and *P* value range r = 0.388–0.464, *P* = 0.002–0.012). The vertical NAT score at 6 months correlated with the nasal RD% at 6 months and the horizontal NAT score at 6 months correlated with the nasal RD% at 1 month and 6 months.

**Table 3:** Correlations of new aniseikonia test score and the retinal displacement.

	2 weeks				1 month				3 months				6 months			
	N	T	S	I	N	T	S	I	N	T	S	I	N	T	S	I
Preoperative																
NATV																
r	− 0.008	**0.464**	0.076	0.281	0.086	**0.415**	0.048	0.152	− 0.084	**0.388**	− 0.096	0.133	− 0.067	**0.445**	− 0.056	0.203
*P*	0.958	0.002*	0.635	0.075	0.591	0.007*	0.764	0.343	0.604	0.012*	0.551	0.407	0.677	0.004*	0.730	0.202
NATH																
r	− 0.235	0.258	0.258	0.189	− 0.049	0.238	− 0.053	0.095	− 0.212	0.278	− 0.139	0.016	− 0.269	0.251	− 0.109	0.064
*P*	0.139	0.104	0.104	0.237	0.761	0.134	0.740	0.556	0.183	0.079	0.387	0.922	0.089	0.114	0.497	0.689
At 6 months																
NATV																
r	− 0.153	− 0.133	− 0.157	− 0.104	− 0.204	− 0.038	− 0.066	− 0.185	− 0.080	0.013	− 0.007	− 0.109	− **0.356**	− 0.068	− 0.102	− 0.180
P	0.340	0.406	0.327	0.517	0.200	0.814	0.684	0.247	0.617	0.934	0.964	0.497	0.022*	0.672	0.524	0.260
NATH																
r	− 0.228	− 0.196	− 0.297	− 0.169	− **0.321**	− 0.127	− 0.212	− 0.226	− 0.218	− 0.050	− 0.167	− 0.168	− **0.375**	− 0.123	− 0.173	− 0.202
*P*	0.151	0.220	0.059	0.290	0.041*	0.427	0.183	0.156	0.170	0.757	0.297	0.294	0.016*	0.445	0.278	0.205
Difference at 6 months																
NATV																
r	− 0.119	**− 0.488**	− 0.208	− 0.270	− 0.227	**− 0.370**	− 0.101	− 0.160	0.003	− 0.304	0.023	− 0.126	− 0.218	**− 0.387**	− 0.079	− 0.269
*P*	0.457	0.001*	0.191	0.088	0.154	0.017*	0.531	0.317	0.986	0.053	0.887	0.434	0.171	0.012*	0.622	0.089
NATH																
r	0.035	**− 0.323**	**− 0.339**	− 0.283	–0.221	− 0.254	− 0.120	− 0.209	− 0.024	− 0.215	− 0.084	− 0.136	− 0.081	− 0.253	− 0.123	− 0.226
*P*	0.829	0.039*	0.030*	0.073	0.166	0.110	0.455	0.191	0.883	0.177	0.603	0.398	0.613	0.110	0.442	0.155

Significant values are in [bold].

*N* nasal retinal displacement %, *T* temporal retinal displacement %, *S* superior retinal displacement %, *I* inferior retinal displacement %, *NATH* horizontal score of new aniseikonia test, *NATV* vertical score of new aniseikonia test.

Significant *P* values are indicated by asterisk (*).

The difference in vertical NAT scores at 6 months correlated with the temporal RD% at 2 weeks, 1 month and 6 months (r and *P* value range r = 0.388–0.464, *P* = 0.002–0.012). The improvement of horizontal NAT scores at 6 months correlated with the temporal and superior RD% at 2 weeks (r = − 0.323, *P* = 0.039 and r = − 0.339, *P* = 0.030).

### Correlations of the NAT scores with changes of retinal layer thickness

Preoperative vertical and horizontal NAT scores correlated with temporal INL% at 2 weeks and 1 month (r and *P* value range r = 0.343–0.411, *P* = 0.008–0.028), and preoperative vertical NAT score correlated with inferior INL% at 2 weeks (r = 0.405, *P* = 0.009) and temporal INL% at 6 months (r = 0.340, *P* = 0.030). Preoperative vertical NAT scores correlated with nasal OR% at 1 month (r = 0.530, *P* = 0.010), as well as horizontal NAT scores with inferior OR% at 2 weeks and 1, 3 months (r and *P* value range r = 0.371–0.457, *P* = 0.003–0.017).

Improvement of vertical NAT scores at 6 months correlated with INL% in the 4 sectors at 2 weeks (nasal: r = − 0.356, *P* = 0.022, temporal: r = − 0.582, *P* < 0.001, superior: r = − 0.422, *P* = 0.006 and inferior: r = − 0.448, *P* = 0.003), with that in temporal and inferior sectors at 1 month (r = − 0.560, *P* < 0.001 and r = − 0.485, *P* = 0.001), with that in the superior at 3 months (r = − 0.440, *P* = 0.004), and with the nasal OR% at 1, 3, and 6 months (r and *P* value range r = − 0.451 to − 0.342, *P* = 0.003–0.028). Improvement of horizontal NAT scores at 6 months correlated with INL% in temporal and superior sectors at 2 weeks, with that in temporal and inferior at 1 month, and with that in inferior at 3 months (r and *P* value range r = − 0.457 to − 0.310, *P* = 0.003–0.049).

In other words, the larger the postoperative change of INL in 4 sectors, especially in the temporal sector, and the larger the postoperative change of OR in nasal sector, the greater the improvement of vertical aniseikonia after MH surgery. Furthermore, the larger the change of INL in 3 sectors except for the nasal, the greater the improvement in horizontal aniseikonia after surgery.

### Correlations of age with retinal displacement

Age correlated with the temporal and inferior RD% at 2 weeks (r = 0.308, *P* = 0.049 and r = 0.316, *P* = 0.044) and the nasal and inferior at 6 months (r = 0.474, *P* = 0.002 and r = 0.312, *P* = 0.047), suggesting that the older the patient, the smaller retinal displacement.

### Multivariate analysis to identify factors associated with NAT scores

The results of stepwise multiple regression analysis to identify predictors for NAT scores are shown in Tables 4 and 5. The dependent factor was the average of the vertical and horizontal NAT scores at baseline, 6 months and differences at 6 months postoperatively. The independent factors were age, sex, MHD, preoperative INL and OR, INL%, OR%, and RD% at 6 months. Preoperative retinal thicknesses, changes of retinal thickness and RD% were analyzed for each of the four sectors. The average of the vertical and horizontal basal MHDs was used for the analysis.

**Table 4:** Multivariate regression analysis for preoperative new aniseikonia test scores.

	Coefficient	SE	t	*P*	95%CI
Dependent factor: preoperative NAT					
Independent factors on nasal sector					
Basal MH diameter	− 0.435	0.002	− 2.734	0.010	− 0.008 to − 0.001
Independent factors on temporal sector					
Basal MH diameter	− 0.407	0.001	− 2.781	0.008	− 0.006 to − 0.001
Independent factors on superior sector					
Basal MH diameter	− 0.473	0.002	− 3.038	0.005	− 0.009 to − 0.002
Independent factors on inferior sector					
Inferior OR %	0.419	0.026	2.885	0.006	0.022 to 0.125
Dependent factor: postoperative NAT at 6 months					
Independent factors on nasal sector					
Age	− 0.318	0.040	− 2.064	0.046	− 0.164 to − 0.002
Independent factors on temporal sector					
Age	− 0.367	0.041	− 2.462	0.018	− 0.183 to − 0.018
Independent factors on superior sector					
Age	− 0.357	0.048	− 2.193	0.035	− 0.203 to − 0.008
Independent factors on inferior sector					
Age	− 0.367	0.041	− 2.462	0.018	− 0.183 to − 0.018

Independent factors: age, sex, basal MH diameter = average of vertical and horizontal basal MH diameter, preoperative INL, preoperative OR, RD%, INL%, OR% at 6 months.

*NAT* new aniseikonia test score, *SE* standard error, *CI* confidence Interval, *MH* macular hole, *INL* inner nuclear layer thickness, *OR* outer retinal thickness, *RD%* rate of change in retinal displacement, *INL%* rate of change in inner nuclear layer thickness, *OR%* rate of change in outer retinal thickness.

**Table 5:** Multivariate regression analysis for difference in new aniseikonia test scores at 6 months.

	Coefficient	SE	t	*P*	95%CI
Independent factors on nasal sector					
Basal MH diameter	0.377	0.001	2.544	0.015	0.001 to 0.006
Independent factors on temporal sector					
Basal MH diameter	0.428	0.001	2.918	0.006	0.001 to 0.006
Age	− 0.333	0.044	− 2.181	0.035	− 0.184 to − 0.007
Independent factors on superior sector					
Basal MH diameter	0.428	0.001	2.918	0.006	0.001 to 0.006
Age	− 0.333	0.044	− 2.181	0.035	− 0.184 to − 0.007
Independent factors on inferior sector					
Basal MH diameter	0.428	0.001	2.918	0.006	0.001 to 0.006
Age	− 0.333	0.044	− 2.181	0.035	− 0.184 to − 0.007

Independent factors: age, sex, basal MH diameter = average of vertical and horizontal basal MH diameter, preoperative INL, preoperative OR, RD%, INL%, OR% at 6 months.

*SE* standard error, *CI* confidence Interval, *MH* macular hole, *INL* inner nuclear layer thickness, *OR* outer retinal thickness, *RD%* rate of change in retinal displacement, *INL%* rate of change in inner nuclear layer thickness, *OR%* rate of change in outer retinal thickness.

The significant predictors for the preoperative NAT score were the basal MHDs and inferior OR%, and that for the postoperative NAT score at 6 months were age. MHD and age were significant predictors for improvement of NAT scores at 6 months. The older the patient, the poorer improvement of aniseikonia and the greater postoperative micropsia.

## Discussion

Our results showed that aniseikonia improved with time after MH surgery. Preoperative aniseikonia correlated with MHD, and improvement of postoperative aniseikonia also correlated with MHD. Preoperative aniseikonia and improvement of postoperative aniseikonia correlated with postoperative temporal retinal displacement. Improvement of postoperative vertical aniseikonia correlated with changes of temporal INL and nasal OR, indicating that the larger postoperative thinning of temporal INL and nasal OR, the greater the improvement of vertical aniseikonia. MHD were predictors for preoperative aniseikonia. MHD and age were predictors for the improvement of aniseikonia.

Recently, the relationship between aniseikonia and retinal diseases such as ERM^9–15^, MH^16^, RRD^17,18^, and branch retinal vein occlusion (BRVO)^19^ has been reported. Okamoto et al.^16^ reported that 55% of MH patients had micropsia and that the degree of preoperative aniseikonia was related to the defect length of the external limiting membrane and diameter of the MH, while postoperative aniseikonia at 12 months did not correlate with any preoperative parameters. Our results that early postoperative aniseikonia correlates with MHD are different from the previous report^16^ probably due to the difference of the times of examination of aniseikonia. Because reconstruction of retinal tissues continues after surgery, aniseikonia shortly after surgery is affected by the size of MH.

Contraction of the inner retina of ERM causes centripetal migration of retinal photoreceptor cells, resulting in aniseikonia, which is mainly perceived as macropsia. The reason why contraction of the inner retina causes metamorphopsia and aniseikonia remains to be elucidated however a reasonable explanation is that if the inner retinal layer is displaced from the photoreceptor layer, Müller cells, which act as optic fibers to transfer photons from the retinal surface to the photoreceptor cells^20,21^, reach the photoreceptor cells far from the original location^9,22^. Following closure of the MH, changes of INL correlated with changes in aniseikonia, and retinal displacement caused INL thinning in eyes with larger MH, indicating that dislocated Müller cells play a significant role in the generation of aniseikonia.

In addition to dislocated Müller cells, the density of photoreceptor cells seems to be critical for generation of metamorphopsia and aniseikonia. As discussed in our previous publication, simultaneous stimulation of the overlapping photoreceptors which were elevated from retinal pigment epithelium may cause a sense of distortion and micropsia in MH^8^.

Earlier, we reported that the degree of aniseikonia significantly correlated with the distance of retinal displacement after ERM surgery^9^, although aniseikonia was unlikely to improve^9,10^. Prolonged persistent macular traction by ERM disrupts the alignment of Müller cells and photoreceptor cells, resulting in permanent changes that are less likely to become normal after surgery^23^, causing the perception of residual aniseikonia after surgery. On the other hand, metamorphopsia and aniseikonia in MH improve in many cases postoperatively, although patients with large MH still complain of them even after successful surgery.

In our present analysis, we found that improvement of aniseikonia was poorer with older age, and that postoperative retinal displacement was smaller with older age. Metamorphopsia is less likely to improve in older patients with ERM as shown in our recent publication^24^. In rats, Müller cells were reported to respond to age-related photoreceptor degeneration by increasing expression of glial fibrillary acidic protein, the intermediate filament protein^25^. These findings suggest that, in humans also, the retina becomes less flexible with age, and consequently recovery of visual function is likely to be delayed.

**Figure 3:**
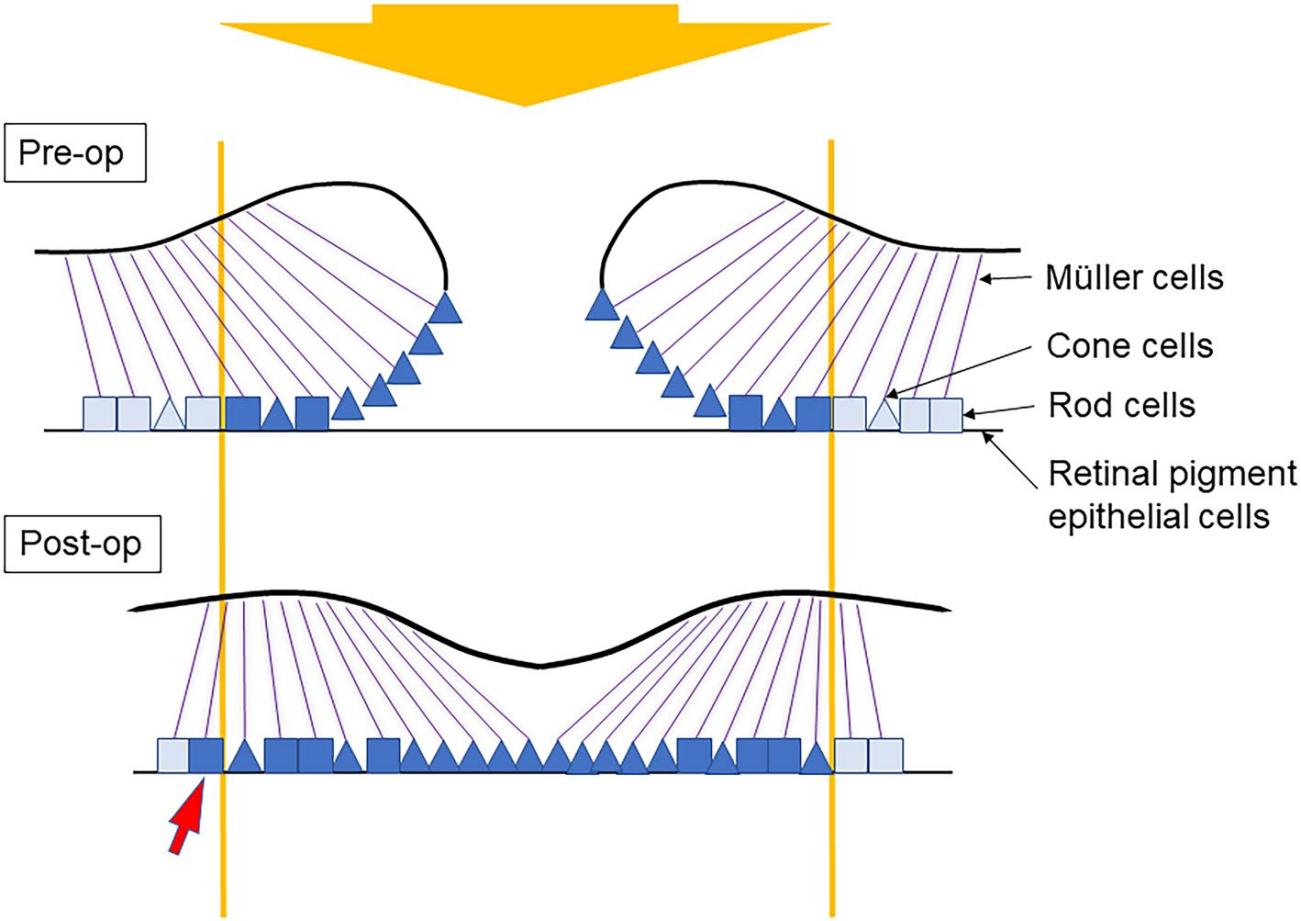
Schematic diagram showing the mechanism of aniseikonia before and after macular hole surgery. The density of photoreceptor cells stimulated by incoming light (yellow arrows) differs between fellow eyes and eyes of the macular hole (MH), resulting in preoperative micropsia. The photoreceptor cells move afferently during closure of the MH and the migration of Müller cells is attributable to thinning of the inner nuclear layer thickness and displacement of the inner retinal layer. When incoming light enters Müller cells, light reaches photoreceptor cells away from the original locations (red arrow). This wide stimulation of the photoreceptor cells may improve sensation of micropsia after surgery.

Interestingly, 17% of patients experienced macropsia after MH surgery. We considered that the presence of macropsia after surgery was due to the afferent migration of the photoreceptor cells around the hole during closure of the MH, and the deviation of Müller cells was attributable to thinning of the INL and displacement of the inner retinal layer (Fig. 3).

The limitations of this study were retrospective study, relatively small number of cases and short follow-up period. The accuracy of the NAT score may be affected by the subject’s visual acuity, and we may need to interpret data from those with low BCVA carefully. Furthermore, areas of ILM peeling were not measured. The possibility of bias due to differences of the area of peeled ILM among surgeons was not completely excluded. The manual measurement points for each retinal thickness were not always identical and changed with tangential migration of the retina after MH surgery.

In conclusion, the aniseikonia in patients with MH improves after surgery. The larger the MHD, the greater the temporal retinal displacement, and the thinner the INL and OR postoperatively, then the greater the improvement of aniseikonia. The changes of photoreceptor density and thinning of the inner retinal layer appear to generate sensations of aniseikonia after MH surgery. In addition to MHD, age is a clinical biomarker for improvement of aniseikonia after MH surgery.

## Data Availability

The datasets generated and analyzed during the current study are available from the corresponding author on reasonable requests.
